# Synthesis, Mesomorphic, and Solar Energy Characterizations of New Non-Symmetrical Schiff Base Systems

**DOI:** 10.3389/fchem.2021.686788

**Published:** 2021-09-03

**Authors:** Fowzia S. Alamro, Hoda A. Ahmed, Sobhi M. Gomha, Mohamed Shaban

**Affiliations:** ^1^ Department of Chemistry, College of Science, Princess Nourah bint Abdulrahman University, Riyadh, Saudi Arabia; ^2^ Department of Chemistry, Faculty of Science, Cairo University, Cairo, Egypt; ^3^ Chemistry Department, College of Sciences Yanbu, Taibah University, Yanbu, Saudi Arabia; ^4^ Chemistry Department, Faculty of Science, Islamic University of Madinah, Almadinah-Almonawara, Saudi Arabia; ^5^ Nanophotonics and Applications Labs, Department of Physics, Faculty of Science, Beni-Suef University, Beni-Suef, Egypt; ^6^ Department of Physics, Faculty of Science, Islamic University of Madinah, Al-Madinah Al-Munawwarah, Saudi Arabia

**Keywords:** lateral methoxy, schiff base liquid crystals, nematic phase, optical properties, electrical properties, solar energy

## Abstract

New asymmetrical Schiff base series based on lateral methoxy group in a central core, (E)-3-methoxy-4-(((4-methoxyphenyl)imino)methyl)phenyl 4-alkoxybenzoate (**An**), were synthesized and their optical and mesomorphic characteristics were investigated. The lateral OCH_3_group was inserted in the central ring in ortho position with respect to the azomethine linkage. FT-IR, and NMR spectroscopy as well as elemental analyses were used to elucidate their molecular structures. Their mesomorphic behaviors were characterized by polarized optical microscopy (POM) and differential scanning calorimetry (DSC). These examinations indicated that all the designed series were monomorphic and possessed nematic (N) mesophase enantiotropically, except **A12** derivative which exhibited monotropic N phase. A comparative study was made between the present investigated series (**An**) and their corresponding isomers (**Bn**). The results revealed that the kind and stability of the mesophase as well as its temperature range are affected by the location and special orientation of the lateral methoxy group electric-resistance, conductance, energy-gap, and Urbach-energy were also reported for the present investigated **An** series. These results revealed that all electrodes exhibit Ohmic properties and electric-resistances in the GΩ range, whereas the electric resistance was decreased from 221.04 to 44.83 GΩ by lengthening the terminal alkoxy-chain to *n* = 12. The band gap of the **An** series was reduced from 3.43 to 2.89 eV by increasing the terminal chain length from *n* = 6 to *n* = 12 carbons. Therefore, controlling the length of the terminal chain can be used to improve the **An** series’ electric conductivity and optical absorption, making it suitable for solar energy applications.

## Introduction

Today, numerous applications are being found for liquid crystals (LCs) due to their ability to undergo molecular orientation changes, such as electromagnetic fields, optical displays, surface modifications, and solar energy applications (Meng et al., 2018; You et al., 2019; Olaleru et al., 2020). On the other hand, the development of LC structural shapes with specific characteristics for certain applications remains a crucial challenge which needs wide information about the correlation between structural shape and mesomorphic properties, as well as their effect on the involved mechanisms of phase transitions (Lagerwall and Giesselmann, 2006).

**Scheme 1:**
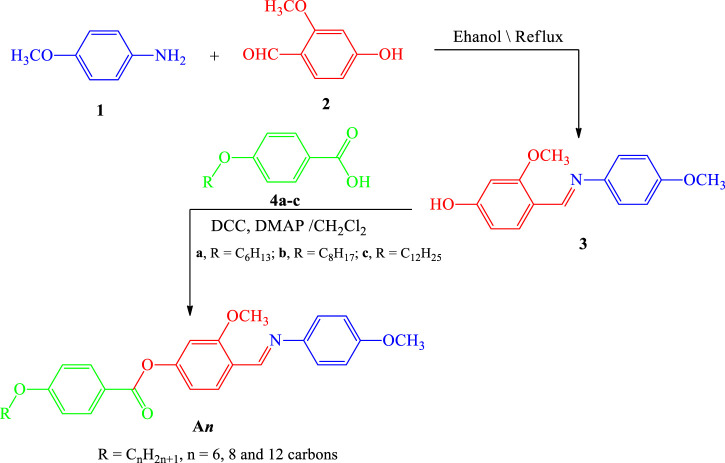
Synthesis way of materials An.

Recently, the small molecule solar cells have exhibited great potential (Badgujar et al., 2016; Bin et al., 2017; Qiu et al., 2017; Bin et al., 2018; Li et al., 2018). Organic solar cells are cost-effective compared to traditional photovoltaic cells. Numerous studies on the applications of organic compounds for photosensitizers in solar cells have been reported (Meng et al., 2018; You et al., 2019; Olaleru et al., 2020). Innovative characteristics of organic solar cells as flexibility, cheap, and ease of use have attracted considerable attention from technological engineers and researchers. Furthermore, modern organic solar cells are low coast and having excellent efficiency (Meng et al., 2018). Due to the applications of solar energy, such as catalytic photo-degradation of dyes, solar hydrogen-generation, photo-electrochemical water splitting, and solar cells, band gap engineering and optical property control are critical parameters of interest (Ahmed and Abdalla, 2020; Helmy et al., 2020; Mohamed et al., 2020; Shaban and El Sayed, 2020; Shaban et al., 2020).

Huge numbers of rod-like thermotropic LCs, with rigid cores containing two or more aromatic rings and terminal flexible chains, have been reported (Kelker and Scheurle, 1969; Sharma and Patel, 2017; Kato et al., 2018). Most of these studies were based on azomethine linkages (Ahmed et al., 2018; Al-Mutabagani et al., 2021; Altowyan et al., 2021; El-atawy et al., 2021). The insertion of high polar compact lateral or terminal groups to main architecture influences the thermal and physical properties of the resulting LC material, such as phase transition temperatures, dielectric anisotropy, and the dipole moment (Jessy et al., 2018; Mishra et al., 2018; Saccone et al., 2018; Zaki et al., 2018; Zaki, 2019). Generally, the intermolecular separation increases due to the addition of lateral substituent, which widens the mesogenic cores and consequently leads to a reduction in lateral interactions (Naoum et al., 1997; Saad and Nessim, 1999; Naoum et al., 2010). However, as the breadth/length of the molecule will increment the thermal stability of produced phases decreases (Luckhurst and Gray, 1979). The small size of the lateral substituent enables its attachment into mesomorphic geometrics without being sterically disrupted, so liquid crystalline mesophases can still be observed. On the other hand, the terminal flexible chain group plays an essential role in the mesomorphic behaviors of synthesized materials (Yeap et al., 2004; Takezoe and Takanishi, 2006). As the length of the flexible terminal chain increases, the molecules tend to be oriented in a parallel alignment (Henderson and Imrie, 2011).

This study aims to synthesize new azomethine derivatives of di-methoxy groups having changeable lengths of the terminal alkoxy-group (n), namely, (E)-3-methoxy-4-(((4-methoxyphenyl)imino)methyl)phenyl 4-alkoxybenzoate, **A*n.***

The methoxy substituent is attached to a Schiff base terminal phenyl linker, while the other CH_3_O group is present into the central of structure as a laterally polar moiety. Moreover, the study aims to investigate the impact of lengthen of alkoxy chain on the mesomorphic properties of synthesized homologues. In addition, a comparison is conducted between the present investigated series and the previously reported isomers to evaluate the impact of exchanging the location of terminal polar groups on the mesomorphic behavior. The research also aims to study their optical and electric behaviors.

## Experimental

### Synthesis

Many reports have revealed that hydrazones and imines are valuable materials for medicinal and synthetic applications (Gomha and Riyadh, 2011; Abu-Melha et al., 2020; Gomha et al., 2020a; Gomha et al., 2020b; Ouf et al., 2020; Sayed et al., 2020; Gomha et al., 2021; Sayed et al., 2021). The following Scheme 1 shows the synthesis of a series of novel lateral CH3O materials **3** and **An**:

Details for synthesis of (E)-3-methoxy-4-(((4-methoxyphenyl)imino)methyl)phenol (3) and (E)-3-methoxy-4-(((4-methoxyphenyl)imino)methyl)phenyl 4-alkoxybenzoate, An are included in the Supplementary Material.

**FIGURE 1:**
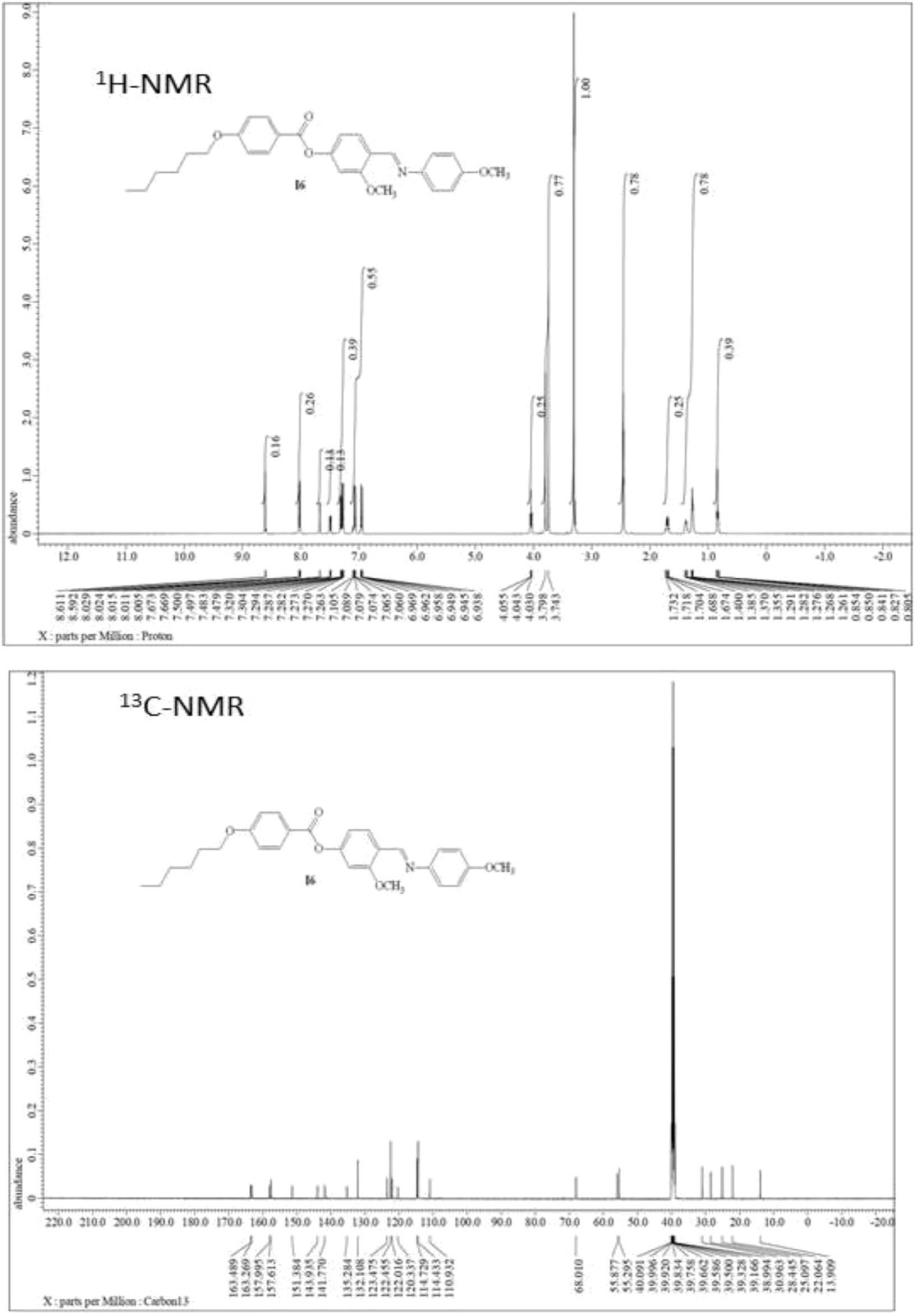
NMR spectra of material **A6**.

**FIGURE 2:**
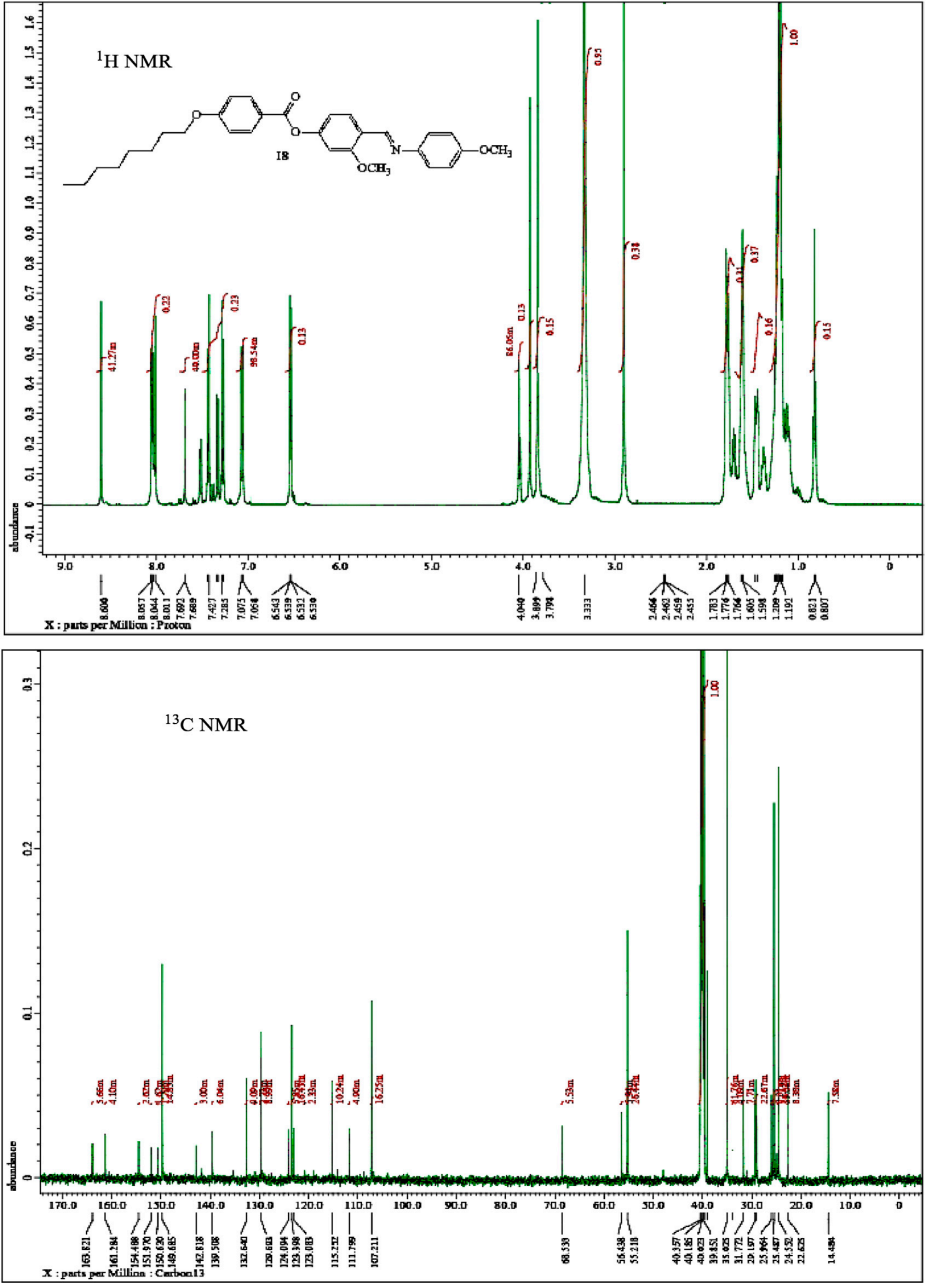
NMR spectra of material **A8**.

**FIGURE 3:**
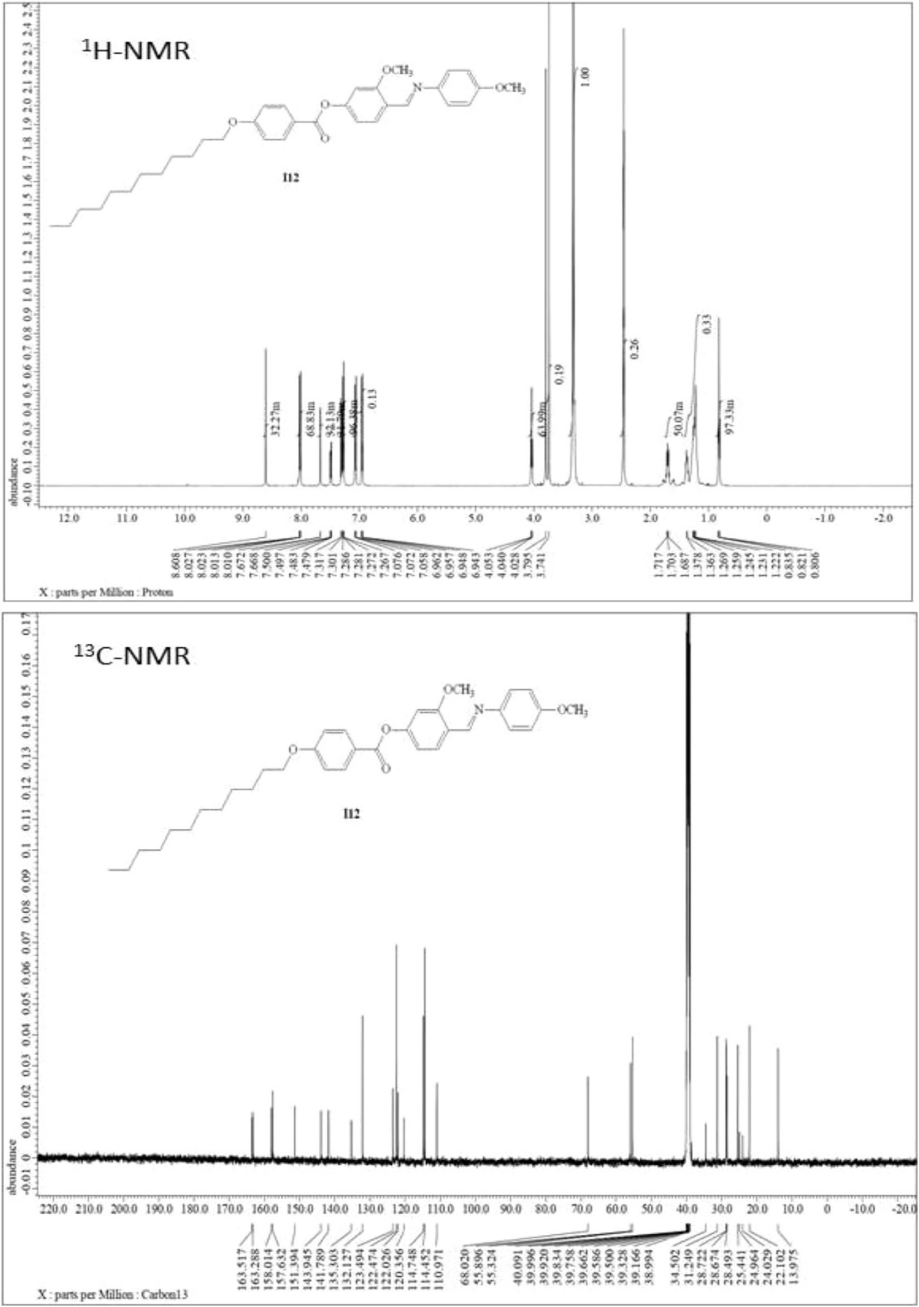
NMR spectra of material **A12**.

^1^H-NMR, ^13^C-NMR, Infrared spectra (IR), and elemental analyses for the investigated materials were in agreement with the assigned structures. ^1^H-NMR data showed the expected ratios (Figure 1, Figure 2, and Figure 3). The physical data of products **A*n*** are listed below:

#### (E)-3-Methoxy-4-(((4-methoxyphenyl)imino)methyl)phenyl 4-(hexyloxy)benzoate (A6)

Yield: 87.3%; mp 103–105 °C, FTIR (ύ, cm^−1^): 3,016, 2,944 (C-H), 1,737 (C=O), 1,622 (C=N). ^1^H-NMR (400 MHz, DMSO): *δ*/ppm: 0.80–0.85 (t, 3H, CH_3_(CH_2_)_3_CH_2_CH_2_O-), 1.26–1.40 (m, 6H, CH_3_ (CH_2_ )_3_CH_2_CH_2_O-), 1.67–1.73 (m, 2H, CH_3_(CH_2_)_3_ CH_2_CH_2_O-), 3.74 (s, 3H, OCH_3_), 3.79 (s, 3H, OCH_3_), 4.03–4.05 (t, 2H, CH_3_(CH_2_)_3_CH_2_ CH_2_O-), 6.93–6.96 (d, 2H, Ar−H), 7.06–7.11(d, 2H, Ar−H), 7.26–7.30 (m, 3H, Ar−H), 7.47–7.50 (d, 1H, Ar−H), 7.66 (s, 1H, Ar−H), 8.00–8.03 (d, 2H, Ar−H), 8.61 (s, 1H, CH = N) ppm; ^13^C-NMR (400 MHz, DMSO): *δ*/ppm: 13.90 (CH_3_), 22.06, 25.09, 28.44, 30.96 (CH_2_), 55.29, 55.87 (OCH_3_), 68.01 (CH_2_-O), 110.93, 114.43, 114.72, 120.33, 122.01, 122.45, 123.47, 132.10, 135.28, 141.77, 143.93, 151.38, 157.61 (Ar-C), 157.99 (C=N), 163.26 (Ar-C-OR), 163.49 (C=O) ppm. Anal. Calcd. for C_28_H_31_NO_5_ (461.55): C, 72.86; H, 6.77; N, 3.03. Found: C, 72.73; H, 6.61; N, 2.93%.

#### (E)-4-(((4-Methoxyphenyl)imino)methyl)-3-methoxyphenyl 4-(octyloxy)benzoate (A8)

Yield: 89.7%; mp 96–97°C, FTIR (ύ, cm^−1^): 3,038, 2,929 (C-H), 1733 (C=O), 1,613 (C=N). ^1^H-NMR (400 MHz, DMSO): *δ*/ppm: 0.80–0.82 (t, 3H, CH_3_(CH_2_)_5_CH_2_CH_2_O-), 1.19–1.60 (m, 10H, CH_3_(CH_2_)_5_CH_2_CH_2_O-), 1.76–1.78 (m, 2H, CH_3_(CH_2_)_5_ CH_2_CH_2_O-), 3.79 (s, 3H, OCH_3_), 3.79 (s, 3H, OCH_3_), 4.01–4.04 (t, 2H, CH_3_(CH_2_)_5_CH_2_ CH_2_O-), 6.53–6.54 (d, 2H, Ar−H), 7.06–7.08 (d, 2H, Ar−H), 7.28–7.32 (m, 3H, Ar−H), 7.42 (d, 1H, Ar−H), 7.68–7.69 (s, 1H, Ar−H), 8.01–8.06 (d, 2H, Ar−H), 8.60 (s, 1H, CH = N) ppm; ^13^C-NMR (400 MHz, DMSO): *δ*/ppm: 14.48 (CH_3_), 22.62, 24.55, 25.48, 25.96, 29.19, 31.77 (CH_2_), 55.21, 56.43 (OCH_3_), 68.53 (CH_2_-O), 107.21, 111.79, 115.25, 123.08, 123.39, 124.09, 129.69, 132.64, 139.50, 142.81, 149.68, 150.62, 151.97 (Ar-C), 154.48 (C=N), 161.28 (Ar-C-OR), 163.82 (C=O) ppm. Anal. Calcd. for C_30_H_35_NO_5_ (489.60): C, 73.59; H, 7.21; N, 2.86. Found: C, 73.42; H, 7.09; N, 2.68%.

#### (E)-4-(((4-methoxyhenyl)imino)methyl)-3-methoxyphenyl 4-(dodecyloxy)benzoate(A12)

Yield: 86.0%; mp 88–89°C, FTIR (ύ, cm^−1^): 3,018, 2,925 (C-H), 1731 (C=O), 1,608 (C=N). ^1^H-NMR (400 MHz, DMSO): *δ*/ppm: 0.80–0.84 (t, 3H, CH_3_(CH_2_)_9_CH_2_CH_2_O-), 1.22–1.36 (m, 18H, CH_3_(CH_2_)_9_CH_2_CH_2_O-), 1.69–1.72 (m, 2H, CH_3_(CH_2_)_9_ CH_2_CH_2_O-), 3.74 (s, 3H, OCH_3_), 3.79 (s, 3H, OCH_3_), 4.02–4.04 (t, 2H, CH_3_(CH_2_)_9_CH_2_CH_2_O-), 6.95–6.96 (d, 2H, Ar−H), 7.05–7.07(d, 2H, Ar−H), 7.26–7.32 (m, 3H, Ar−H), 7.48–7.50 (d, 1H, Ar−H), 7.68 (s, 1H, Ar−H), 8.01–8.03 (d, 2H, Ar−H), 8.61 (s, 1H, CH = N) ppm; ^13^C-NMR (400 MHz, DMSO): *δ*/ppm: 13.97 (CH_3_), 22.10, 24.02, 24.96, 25.44, 28.49, 28.67, 28.72, 31.24, 34.50 (CH_2_), 55.32, 55.89 (OCH_3_), 68.02 (CH_2_-O), 110.97, 114.45, 114.74, 120.35, 122.02, 122.47, 123.49, 132.12, 135.30, 141.78, 143.94, 151.39, 157.63 (Ar-C), 158.01(C=N), 163.28 (Ar-C-OR), 163.51 (C=O) ppm. Anal. Calcd. for C_34_H_43_NO_5_ (545.71): C, 74.83; H, 7.94; N, 2.57. Found: C, 74.71; H, 7.84; N, 2.39%.

## Results and Discussion

### Mesomorphic Investigations of Present Series, An

**FIGURE 4:**
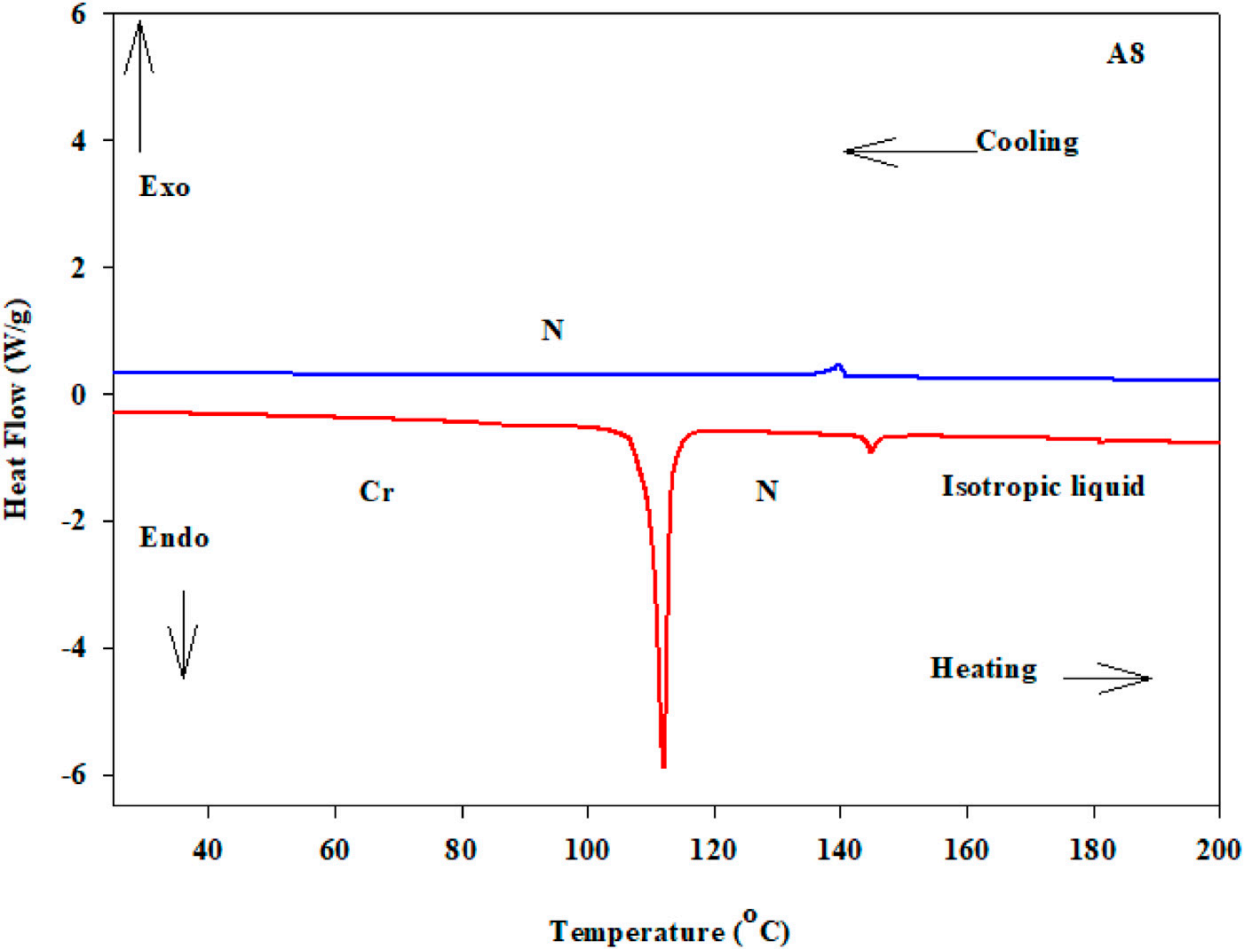
DSC thermograms of derivative **A8** with heating /cooling rate of ±10°C/min.

**FIGURE 5:**
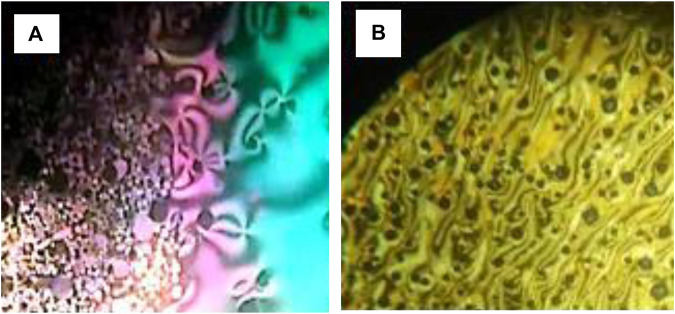
Nematic phase textures upon heating observed under POM for compounds **(A) A6** at 155.0°C and **(B) A10** at 115.0°C.

**FIGURE 6:**
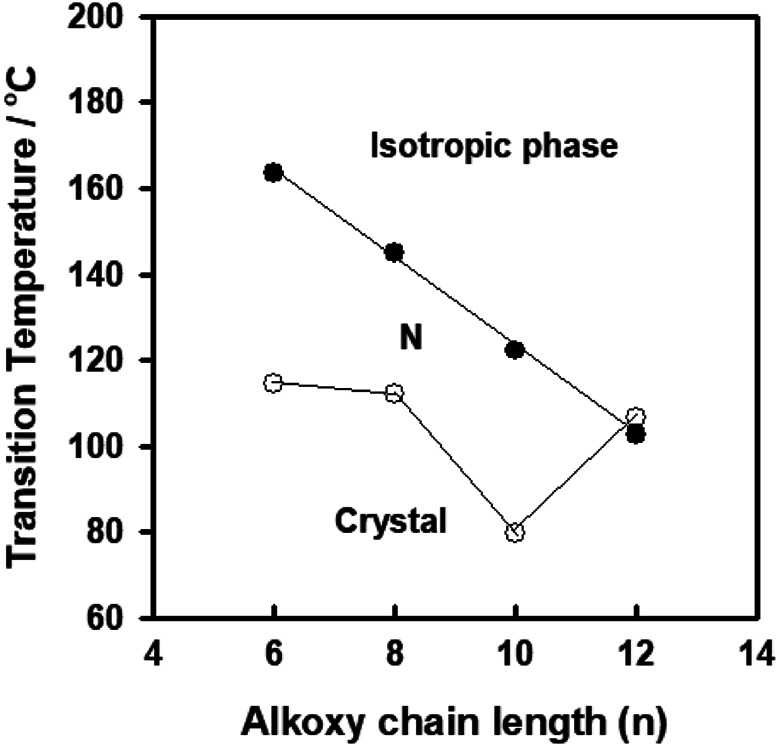
Effect of terminal alkoxy-group on the mesophase behavior of the series **An**.

The mesophase characteristics of the synthesized have been investigated via POM and DSC. Figure 4 shows representative DSC thermograms of homologue **A8** upon heating and cooling cycles. It was observed that the phase transitions from Cr→ N, and N→ I on heating and reversed on cooling for the short chain length **A6** derivative. Transition peaks changed according to the molecular geometry of the designed materials, **An**. Significant endothermic and exothermic peaks were observed to be dependent on the length of the terminal alkoxy chain (n), and were ascribed to mesomorphic transition. Optical images of **A6** and **A10** derivatives under POM are depicted in Figure 5. Schlieren/threads textures of the nematic phase were identified upon heating and cooling scans. The mesomorphic transition temperatures, as derived from DSC evaluations, and their associated enthalpies for all the synthesized compounds, **An**, are summarized in Table 1. The impact of the terminal length of the attached flexible group on their mesomorphic properties is displayed in Figure 4. Results in Table 1 and Figure 6 show that all investigated members of the group **An** are monomorphic and possess enantiotropic N phase, except the longer chain compound **A16** which is monotropic nematogenic. In addition, the homologues **An** series exhibit a wide nematogenic range and stability dependent on their terminal chain length, where the **A16** derivative has the lowest nematic stability. The melting transition of the present compounds, as usual, varies irregularly with the terminal chain length (n). From Figure 6, the shortest chain length derivative **(A6**) exhibits the highest nematic thermal stability and temperature range 163.6 and 49.1 °C, respectively. The A8 sample also possesses N phase enantiotrpoically with N stability and range nearly 144.9 and 32.8°C, respectively. Moreover, the derivative **A10** has the lowest melting temperature 79.8°C, and possesses less enantiotropic thermal nematic stability (122.3°C). The compound bearing the longest chain terminal length (**A12**) has the lowest thermal nematic stability, so its phase appears monotropically. The geometry, polarizability, and the dipole moment of the designed materials are profoundly affected by the mesomeric kind of the terminals. In expansion, the mesomorphic character is impacted by an increase within the polarity and/or polarizability of the mesogenic part. Moreover, the decrement in N stability with the increasing length of the terminal chains (Figure 6) is associated with the increment of the dilution of interactions within the mesogenic units as well as the increment of the volume fraction of the alkoxy chains (Walker et al., 2019). The nematic range of the present series decreases in the order: **A6** > **A10** > **A8** > **A12**. The phase character of calamitic molecules is specifically affected by molecular‐molecular interactions that mainly depend on their shapes and the location of the polar lateral and terminal attached groups.

**TABLE 1:** Temperatures of mesomorphic transitions, °C (enthalpy **ΔH**, kJ/mole), mesophase range (**ΔT**, °C), and the normalized-entropy, **ΔS**/R, of transition for investigated series **An**.

Comp	*T*_Cr-N_	*ΔH*_Cr-N_	*T*_N-I_	*ΔH*_N-I_	*ΔT*	*ΔS*_N-I_/R
A6	114.5	48.43	163.6	1.15	49.1	0.32
A8	112.1	46.06	144.9	1.61	32.8	0.46
A10	79.8	56.05	122.3	1.04	42.5	0.32
A12	106.5	53.94	102.8^a^	1.02	3.7	0.33

Cr-N = solid to the nematic mesophase transition.

N-I = nematic to the isotropic liquid mesophase transition.

a Monotropic phase.

The normalized entropy changes, ΔS_N-I_/R, of the present investigated series (**An**) are summarized in Table 1. The data indicated that independent of the terminal alkoxy chains length, the entropy of N-I transitions show small values with irregular trends that mainly depend on the type of terminal and lateral substituents. Their relatively lower values may be due to the formation of molecular biaxiality (Henderson et al., 2001; Chan et al., 2012; Lee et al., 2012). These results are inconsistent with the previous investigations for dimeric LC materials based on pyrene derivatives (Attard et al., 1992; Attard and Imrie, 1992). Also, the stereo configuration of the lateral methoxy group plays an essential role in the molecular separations. Furthermore, the thermal cis-trans isomerization of the Schiff base linker has an essential role in the observed lower entropy changes, as reported before (Attard et al., 1990; Imrie et al., 1993; Henderson et al., 2001).

### Effect of Position of Lateral Methoxy Group in the Mesomorphic Properties

To investigate the effect of the location of lateral CH_3_O groups on the phase and thermal properties of the materials, a comparison was made between the presently investigated series **An** and their previously corresponding isomers **Bn** (Vora and Gupta, 1982) for their mesomorphic properties. The comparison indicated that the thermal stability of the produced phase varies according to the improved molecular dipole moment and polarizability of the lateral methoxy group, which are dependent upon their position. The mesomorphic properties are nearly the same for the shortest terminal chain derivatives (*n* = 6 and *n* = 8) for both groups, while the longest chain compounds **B10** and **B12** have higher thermal stability than **A10** and **A12**, respectively. It could be concluded that the observed nematic range and stability depend on the location and special orientation of the lateral CH_3_O moiety which was inserted in the mesogenic molecular part.

### Electric Properties

**FIGURE 7:**
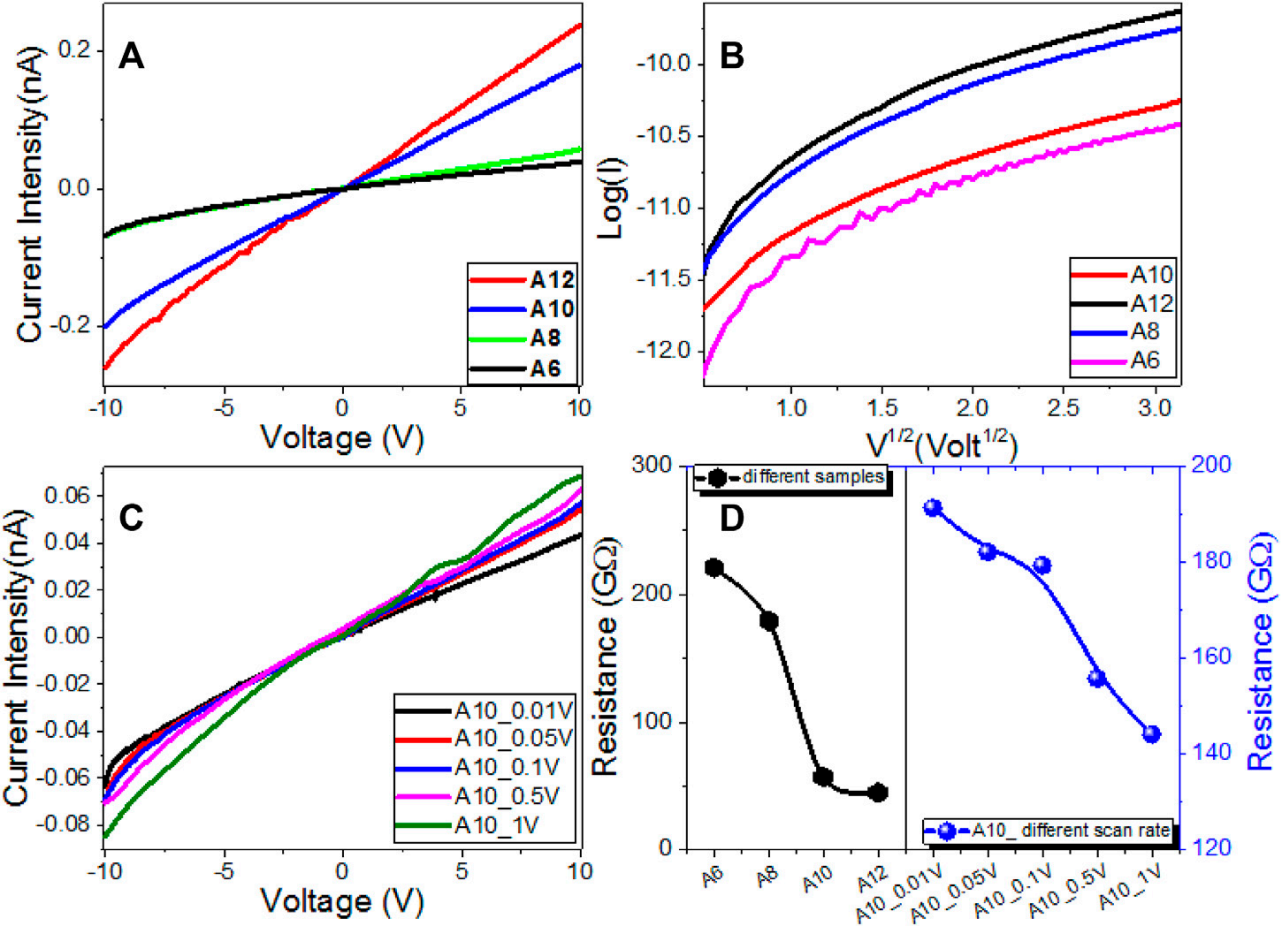
Electrical characteristics of **An** series: **(A)** Current-Voltage characteristics of **An** series, **(B)** Log(I) vs. V^0.5^ For S10 sample at different step scans, **(C)** Current-Voltage characteristics of **A10** sample at different step scans, and **(D)** electric resistance for the **An** samples and **A10 at** different step scans.

The investigated **An** series’ electrical properties and current-voltage (I–V) characteristics are measured from −10 to 10 V at different scan steps; 1.0, 0.5, 0.1, 0.05, and 0.01 V; and shown in Figures 7A,C. The trends are almost linear (Ohmic behaviors). As a consequence, the resistances of the **An** electrodes are almost constant and unaffected by the current passing through them. Polymeric and organic systems act like Schottky diodes at low voltage, according to recent research. However, as shown in Figure 7B, the relationship between log (I) and V^1/2^ is non-linear in the current study, implying that our **An** electrodes do not behave like Schottky diodes. Figure 7A shows how increasing the applied voltage and increasing the terminal alkoxy-chain length to 12 increased the current intensity. The current intensity for the **An** series increased to 0.24 nA@10V when the applied voltage was increased to 10 V and the terminal alkoxy-chain length was increased to 12. As the scan step increased from 0.01 to 1 V, the current intensity is slightly increased, Figure 7C. The resistance of the **An** series is decreased by increasing the terminal alkoxy-chain length to 12. The values of the resistance are decreased from 221.04 GΩ for **A6** to 44.83 GΩ for **A12**. The electric resistance of **A10** film is decreased from 191.42 to 144.13 GΩ by increasing the scan step from 0.01 to 1 V as shown in Figure 7D. The values of the electric conductance (σ) were obtained and shown in Supplementary Figure S2 (Supplementary Material) and Table 2. The value of the electrical conductance is increased from 4.52 pS to 22.3 pS by increasing the terminal alkoxy-chain length from *n* = 6 to *n* = 12 carbons, as shown in Table 2, since the electrical conductance depends mainly on the number and mobility of charge carriers (Rathi et al., 2017; Kumar et al., 2018). By increasing the scan step from 0.01 to 1 V, the film conductance is increased from 5.22 to 6.94 pS. This indicates the coherent photocurrent generation, which is the basis of the photovoltaic cell (Bian et al., 2020).

**TABLE 2:** Values of the electric conductance,**σ,** energy gap, Eg, and Urbach energy, *E*_*U*_, of **An** series.

Sample	σ (pS)	Eg (eV)	EU (meV)	SD	R^2^
A12	22.31	2.89	251.3	3.11	0.9983
A10	17.54	2.91	839.4	8.64	0.9965
A8	5.57	3.01	150.2	2.46	0.9973
A6	4.52	3.43	1,065.0	9.84	0.9975

### Optical Spectra and Energy Gap Calculation

**FIGURE 8:**
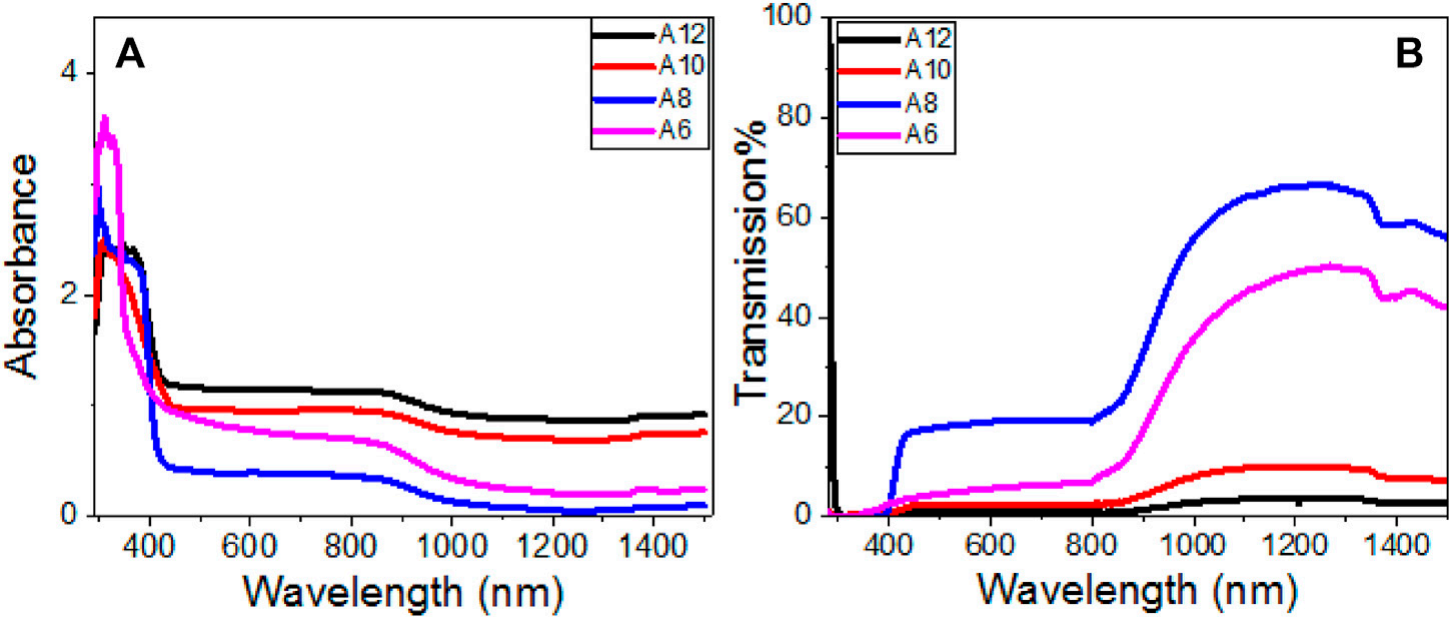
Optical **(A)** absorbance and **(B)** transmittance spectra of **An** films.

The wavelength of incident light and the length of the **An** series’ terminal alkoxy-chain influence the transmittance and absorbance spectra of the **An** series, as shown in Figures 8A,B. All films showed transmission close to zero up to 400 nm, then the transmission increased and became less than 20% for **An** samples in the visible light region, Figure 8A. The transmission increased exponentially in the near IR region to reach maxima of ∼50, 66, 10, and 4% at 1,244 nm for **A6**, **A8**, **A10**, and **A12** electrodes. After that, the transmission decreased as the wavelength increased. The absorbance spectra in Figure 8B show that **An** has strong absorption behavior in the UV/Vis region up to ∼860 nm. For the present **An series,** all films displayed very strong absorbance in the UV region up to ∼400 nm and the strongest absorbance was observed for **A6** and the widest band was observed for **A8** *.* The absorbance then dropped to a plateau from 400 to 860 nm, then dropped again to a minimum absorbance of around 1,250 nm. Figure 8B shows strong and wide absorption bands centered at ∼341.6, 340.4, 333.6, and 315.5 nm for **A12**, **A10**, **A8**, and **A6**, respectively, which is blue-shifted by decreasing the terminal alkoxy-chain length of the prepared **An** series. The bandwidths of these absorption bands are 39.2 nm for **A12**, 101.9 nm for **A10**, 112.9 nm for **A8**, and 54.8 nm for **A6**. The right edge of the absorption band is red-shifted by increasing the terminal alkoxy-chain length in the **An** series. This red-shift is mainly attributed to the size effects, where large size increases spin-orbit coupling and controls the exciton positions (Shaban and El Sayed, 2016). The absorption in the visible and IR region is in the order **A12 > A10 > A8**. This strong absorption and wide absorption band in the visible region is a desirable feature for the designing of energy-efficient solar cells (Liu et al., 2016).

According to the optical absorption theorem, the relationship between absorption coefficient, *α*_*a*_ *,* and the photon energy, *E*_*ph*_ = *hν, h =* 6.625x10^−34^ J/s*,* for the direct allowed transition is given by (Shaban and El Sayed, 2015):

(αa Eph)2 = A( Eph - Eg ) (1)

**FIGURE 9:**
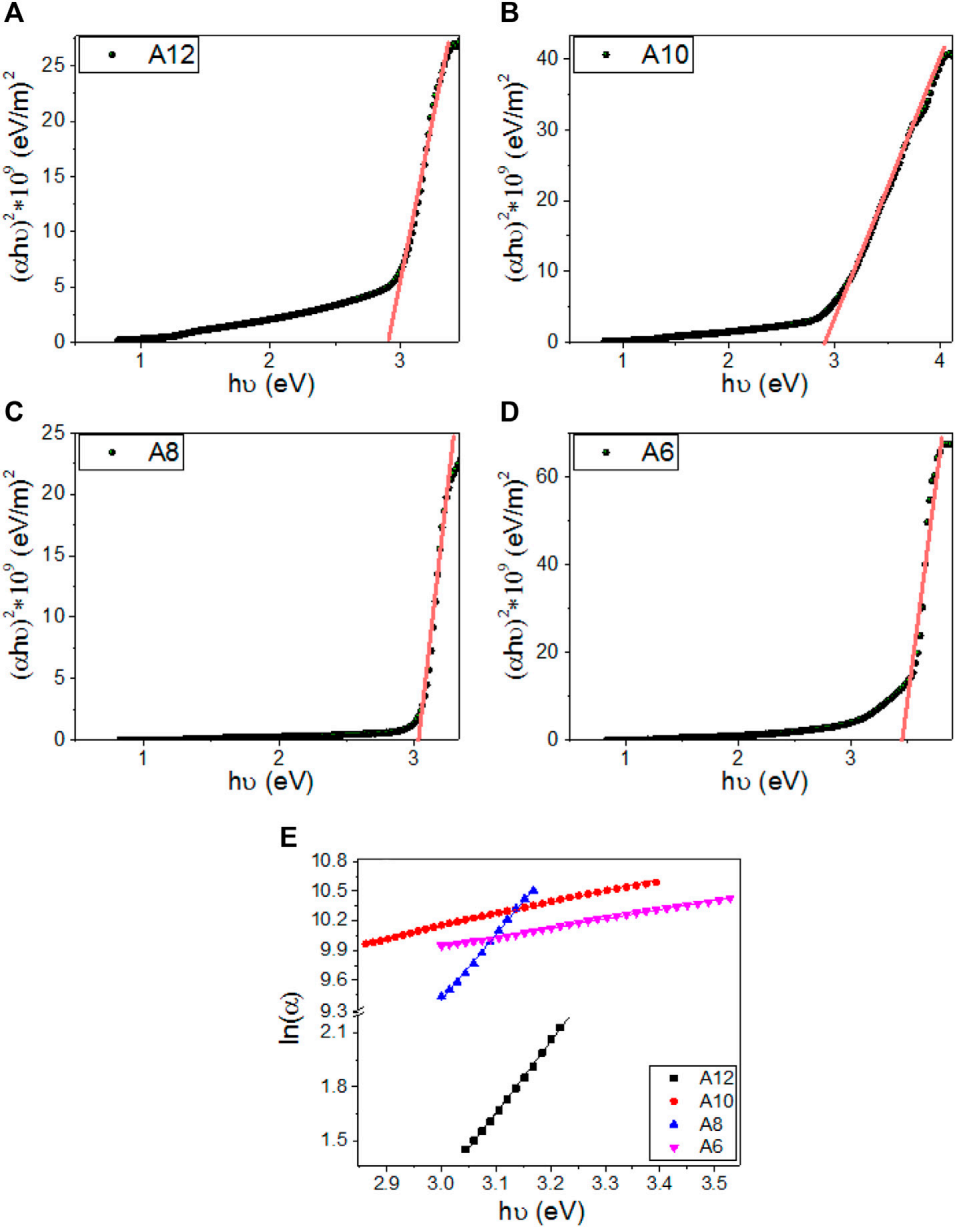
Calculation of energy gap for **(A) A12**, **(B)** **A10**, **(C) A8**, **(D) A6** derivatives, and **(E)** calculation of Urbach energy for **An** series.

Where *E*_*g*_ is the optical energy gap. The values of direct *E*_*g*_ for **A12**, **A10**, **A8**, and **A6** are obtained by extending the linear segments of the plot of (*α*_*a*_ *E*_*ph*_)^2^ vs. *E*_*ph*_ to *zero* as shown in Figure 9A–D. The linear part observed for this figure indicates that the transition is performed directly. Interestingly as reported in Table 2, there is one direct bandgap for each electrode. The value of the bandgap is decreased from 3.43 to 2.89 eV by increasing the terminal chain length from six carbons (**A6**) to 12 carbons (**A12)**. This reduction in the energy gap is ascribed to the influence of the density of localized states and is preferred for solar energy applications (Ahmed and Abdalla, 2020; Helmy et al., 2020; Mohamed et al., 2020; Shaban and El Sayed, 2020; Shaban et al., 2020). This behavior is consistent with the previously reported studies (Li et al., 2019). The strong absorption in the Visible/IR region and the extension of the bandgap edges are very important for solar energy applications, especially photoelectrochemical hydrogen generation and solar cells (Abdelmoneim et al., 2021; Mohamed et al., 2021; Shaban et al., 2021).

Urbach energy (*E*_*U*_) refers to the disorder in the material and represents the width of the exponential absorption edge Urbach tail of the valence and conduction bands (El Sayed and Shaban, 2015). The exponential dependency of the *E*_*U*_ can be determined according to the following equation (El Sayed and Shaban, 2015):

$$\alpha_{\text{a}}\;=\;\alpha_{\text{ao}}\text{exp }\left(E_{\mathrm{ph}}{\text{/E}}_{\text{u}}\right)\to E_{u}=\;\delta E_{\mathrm{ph}}\text{ /}\delta \left(\ln\left(\alpha_{\text{a}}\right)\right)$$ (2)

Where *α*_*ao*_ is the band tail parameter that can be given by (Sharma et al., 2014):

$$\alpha_{\text{ao }}={\left(4\pi \;\sigma \text{o / x }\Delta \mathrm{E c}\right)}^{1\text{/}2}$$ (3)

Where *c* is the speed of light, *σ*_*o*_ is electrical conductivity at absolute zero, and *ΔE* represents the width of the tail of the localized state in the forbidden gap. Figure 9E shows the plot of ln(*α*) vs. *hν* for the two band gaps of **A6**, **A8**, **A10**, and **A12**. The values of *E*_*U*_ were obtained from the slopes of the linear fitting of these curves and are reported in Table 2. The statistical parameters, standard deviation (SD) and correlation coefficient (R^2^), are also reported in this table. The values are 251.3 ± 3.11 for **A12** and 1,065.0 ± 9.84 for **A6**, which refers to the extension of the bandgap edges to cover a wide range of the spectral range. The minimum value of *E*_*U*_ was reported for **A8**.

Tetracene (**C**) and pentacene (**D**) are small organic molecule semiconductors and most broadly investigated as p-type conjugated compounds in solar cells with high carrier mobilities of up to 0.1 and 3 cm2V-1 s-1, respectively. Due to their planar conjugated geometrical structures, they have a relatively low band energy gap of 1.7 eV. Thus they are suitable to be used as p-type semiconductors in photovoltaics (Mishra and Bäuerle, 2012).

The compounds being studied (**An**) are dielectrics due to their high resistance and energy band-gap values. In the presence of an external electric field, dielectric materials can store electric energy due to their polarization. Specifically, the dielectric energy-storing devices that allow for faster energy delivery (i.e., a quicker charge or discharge time), and hence can have promising applications on hybrid electric vehicles and power pulse devices. In the future, **An** compounds can be further refined by integrating conductive plasmonic nanomaterials to improve the conductivity and minimize the band-gap, allowing these samples to be utilized in solar energy applications such as solar cells, photoelectric cells, and photo-electrochemical cells.

## Conclusion

New mesomorphic non-symmetrical homologues series based on a lateral CH_3_O group in a central core, (E)-3-methoxy-4-(((4-methoxyphenyl)imino)methyl)phenyl 4-alkoxybenzoate (**An**), were synthesized and investigated for their potential in solar energy applications. Molecular structure elucidation for the series was carried out by elemental analyses, FT-IR, and NMR spectroscopy. Examination of their mesomorphic behaviors was conducted *via* DSC and POM which indicated that all the synthesized homologues members are purely nematogenic and possess enantiotropic N mesophase, except the longer terminal chain compound (**A12**) which exhibited monotropic N phase. A comparative study between the present series (**An**) and their corresponding isomers (**Bn**) revealed that the mesophase stability and kind, as well as its temperature range, are affected by the location and special orientation of the lateral CH_3_O group. Measurements from the solar energy conversion devices showed that all studied **An** series exhibited Ohmic behavior with electric resistances in the GΩ range. The resistance of the **An** series was decreased by lengthening the terminal alkoxy-chain to *n* = 12 carbons. The highest electric conductivity, 22.3 pS, was reported for **A12**. The value of the bandgap was reduced from 3.43 to 2.89 eV by increasing the terminal chain length from *n* = 6 (**A6**) to *n* = 12 (**A12**). The minimum band edge tail, 150.2 ± 2.46 was reported for the **A8** derivative. Therefore, increasing the length of the terminal chain will increase the **An** series’ electric conductivity and optical absorption, making it appropriate for solar energy applications.

## Data Availability

The original contributions presented in the study are included in the article/Supplementary Material, further inquiries can be directed to the corresponding authors.
