# Supplementary material for: Synthesis, Mesomorphic, and Solar Energy Characterizations of New Non-Symmetrical Schiff Base Systems
Source: Front Chem. 2021 Sep 3;9:686788. doi: 10.3389/fchem.2021.686788 (PMC8448195; doi:10.3389/fchem.2021.686788)
Supplement: Supplementary file 1 [file DataSheet1.docx]

**Supplementary Materials**

**Synthesis, mesomorphic and solar energy characterizations of new non-symmetrical Schiff base Systems**

**Fowzia S. Alamro^1^, Hoda A. Ahmed^2,3*^, Sobhi M. Gomha^2,4*^ and Mohamed Shaban^5,6^**

*^1^Department of Chemistry, College of Science, Princess Nourah bint Abdulrahman University, Riyadh 11671, Saudi Arabia,* *fsalamro@pnu.edu.sa*

*^2^Department of Chemistry, Faculty of Science, Cairo University, Cairo 12613, Egypt,* [*ahoda@sci.cu.edu.eg*](mailto:ahoda@sci.cu.edu.eg)*,* [*smgomha@iu.edu.sa*](mailto:smgomha@iu.edu.sa)

*^3^Chemistry Department, College of Sciences, Yanbu, Taibah University, Yanbu 30799, Saudi Arabia.*

*^4^Chemistry Department, Faculty of Science, Islamic University in Almadinah Almonawara, Almadinah Almonawara, 42351, Saudi Arabia*

*^5^ Nanophotonics and Applications Labs, Department of Physics, Faculty of Science, Beni-Suef University, Beni-Suef 62514, Egypt*

*^6^Department of Physics, Faculty of Science, Islamic University in Almadinah Almonawara, Almadinah, 42351, Saudi Arabia*

** Correspondence:* (HA.A) [*ahoda@sci.cu.edu.eg*](mailto:ahoda@sci.cu.edu.eg) and *(SM.G)* [*sm.gomha@iu.edu.sa*](mailto:sm.gomha@iu.edu.sa)

1. ***Materials***

4-Hexyloxybenzoic acid, 4-octoyloxybenzoic acid, 4-dodecyloxybenzoic acid 4-hydroxy-2-methoxybenzaldehyde, and 4-methoxyaniline, were purchased from Sigma Aldrich (Germany). dichloromethane, *N,N'*-dicyclohexylcarbodiimide (DCC), ethanol and 4-dimethylaminopyridine (DMAP) were purchased from Aldrich (Wisconsin, USA).

1. ***Synthesis of (E)-3-methoxy-4-(((4-methoxyphenyl)imino)methyl)phenol (3)***

A mixture of 4−hydroxy-2-methoxybenzaldehyde (1.52g, 10 mmol) and 4-methoxyaniline (1.23g, 10 mmol) in ethanol (20 mL) were refluxed for two hours (monitored by TLC). The mixture was cooled to room temperature and filtered. The obtained solid was washed was cold ethanol and recrystallized twice from hot ethanol to give pure imine compound **3** as indicated by TLC analysis. The melting points and IR data determined of the prepared imine **3** as indicated by TLC analysis. The melting points and IR data determined of the prepared imine **3:** Yield: 92.7%; mp 101.1 ^°^C, FTIR (ύ, cm^−1^): 3431 (OH), 2919, 2842 (CH_2_ stretching), 1735 (C=O), 1618 (C=N), 1571 (C=C), 1460 (C−O _Asym_), 1244 (C-O _Sym_). ^1^H-NMR (400 MHz, CDCl_3_): *δ*/ppm: 3.72 (s, 1H, OH), 3.78 (s, 3H, OCH_3_), 3.81 (s, 3H, OCH_3_), 6.79 (d, 1H, Ar−H), 6.79 (d, 2H, Ar−H), 6.89 (d, 2H, Ar−H), 7.01 (d, 1H, Ar−H), 7.24 (s, 1H, Ar−H), 7.32 (d, 2H, Ar−H), 9.73 (s, 1H, CH=N). Anal. Calcd. for C_15_H_15_NO_3_ (257.28): C, 70.02; H, 5.88; N, 5.44. Found: C, 70.14; H, 5.73; N, 5.29%.

1. ***Synthesis of (E)-3-methoxy-4-(((4-methoxyphenyl)imino)methyl)phenyl 4-alkoxybenzoate, An:***

A mixture of imine compound **3** (2.57g, 10 mmol) and the appropriate 4-alkoxybenzoic acid derivatives **4** (10 mmol for each) in dry methylene chloride (25 mL) containing *N*, *N′−*dicyclohexylcarbodiimide (DCC, 10 mmol) and few crystals of 4*−*dimethylaminopyridine (DMAP), as catalyst, were left to stand for 72 hours at room temperature with continuous stirring. The solid separated was then filtered off and the solution evaporated. The solid residue obtained was recrystallized from ethanol to give TLC pure products. The purity of the prepared samples was checked with thin-layer chromatography (TLC) using TLC sheets coated with silica gel (E Merck), and CH_2_Cl_2_/CH_3_OH (9:1) as eluent, whereby only one spot was detected by a UV-lamp. Infrared spectra (IR), ^1^H-NMR, and elemental analyses for compounds investigated were consistent with the structures assigned.

1. ***Characterization***

Perkin-Elmer B25 (Perkin-Elmer, Inc., Shelton, CT USA) spectrophotometer was used for infrared spectra measurements. Varian EM 350L 500 MHz spectrometer (Oxford, UK) was used for recording ^1^HNMR spectra using tetramethyl silane as internal standard in CDCl_3_; the chemical shift values recorded as δ (in ppm units). Thermo Scientific Flash 2000 CHS/O Elemental Analyzer, Milan, Italy was used for Elemental analyses.

TA Instruments Co. (Q20 Differential Scanning Calorimeter, DSC; USA) was used for recording phase transitions. DSC calibration was carried out using lead and indium to calibrate the melting temperatures and enthalpies. Samples of 2–3 mg were used in aluminum pans for DSC investigation. The heating rate was 10°C/min in nitrogen gas as an inert atmosphere (30 ml/min). All transitions temperatures were measured from the second heating scan.

Transition temperatures for the prepared compounds were checked and phases identified by Polarized optical microscope (POM, Wild, Germany) attached with Mettler FP82HT hot stage.

A Perkin Elmer spectrophotometer (Lambda 950 UV-VIS-NIR) was used to measure the optical absorbance and transmission spectra of the investigated series, **An**, over a wavelength range of 250 to 1500 nm. The electrical properties of the examined films are tested using a Keithley measurement source system (Model 4200 SMU). By varying the applied voltage (V) from -10 V to 10 V with different scan steps of 1V to 0.01V, the current-voltage (I–V) characteristics of the **An** series are reported.


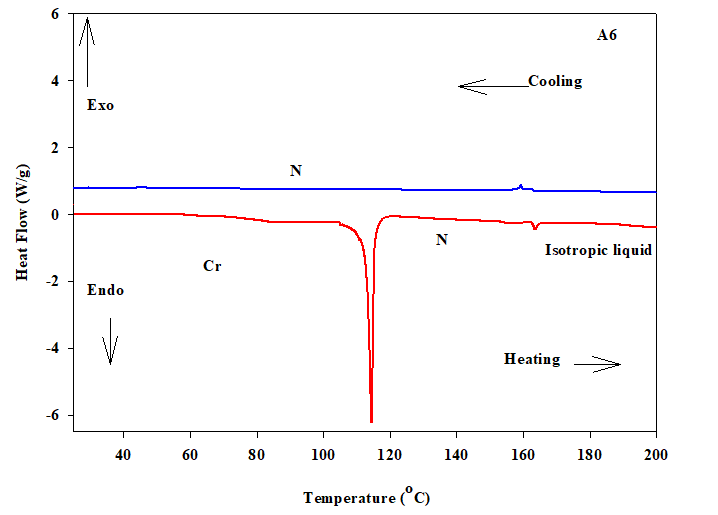


**Figure S1:** DSC thermograms of derivative **A6** at a rate of ±10°C/min which recorded from heating and cooling scans**.**

**Figure S2**: (A) electric conductance for the **An** series and **S10** sample at different step scans.
